# Capturing Gender Identity Through Documentation: A Program Evaluation of a Rural Mental Health Clinic

**DOI:** 10.1111/phn.70039

**Published:** 2025-11-04

**Authors:** Pamela Byrnes, Julie Roebuck

**Affiliations:** ^1^ University of Virginia School of Nursing Charlottesville Virginia USA

**Keywords:** adolescent mental health, documentation, gender identity, program evaluation, public health nursing, rural health, transgender youth

## Abstract

**Background:**

Gender identity documentation is an important component of gender‐affirming care, particularly for LGBTQIA+ adolescents who may be hesitant to disclose their gender identities due to fear of rejection or discrimination. Without family support, these adolescents face higher rates of suicide, depression, and anxiety. Despite both clinical relevance and the importance of providing inclusive health care services, currently, there is no national standard for collecting gender identity information within clinical settings.

**Aim:**

This program evaluation assessed gender identity documentation practices for adolescents in a rural mental health clinic and examined compliance with the American Academy of Pediatrics (AAP) recommendations for inclusive care.

**Methods:**

A retrospective chart review of 151 patients aged 4–18 was conducted from 2020–2023 to evaluate baseline and longitudinal documentation of gender identity and pronouns.

**Results:**

Gender identity documentation improved from 0% in 2020 to 95% in 2023. Pronoun documentation remained inconsistent, and identity was assessed only at intake, limiting the ability to support adolescents with gender identities evolving over time. A case example of a transgender adolescent illustrated how gaps in documentation can contribute to distress and unmet mental health needs.

**Conclusion:**

Systematic gender identity documentation is feasible in a rural outpatient clinic and provides a replicable model for inclusive practice. These findings can guide public health nurses in reducing disparities, influencing policy, and supporting multi‐step efforts to improve care of gender‐diverse youth.

## 1 Introduction

Her heart was pounding as she sat in the waiting room beside her mother, waiting to be called back. Though she knew she needed to be here, she was secretly planning her escape through the side door. Her mother was not quite sure what had changed over the past year, but it was evident in Jamie's mood, behaviors, and academic decline that there had been a shift.

Jamie was a 16‐year‐old female who had spent most of her life feeling out of place, as if living a lie. She knew she was meant to be male yet felt stuck living as a female. Jamie felt scared to talk about this. How could anyone understand? She had never met anyone like herself in her small town—only in movies or on the news had she seen others like her. To feel more like herself, she would dress a bit more masculine and ask her friends to call her Jay—a shorter, more masculine version of her name that would not raise her parents’ suspicions.

The nurse finally called “Jamie,” and she cringed. With her mother, the nurse began the initial intake questions—basic health‐related queries. The nurse asked if she had a different name she would like to be called, and then confirmed her pronouns were she/her. As the nurse went through these questions, Jamie's heart began to race. Was now the time to be honest? However, she did not know this nurse. What would her mom say? What would happen?

## 2 Background

Like Jamie, many adolescents in the LGBTQIA+ community are often hesitant to disclose their gender identity due to fear of rejection or discrimination (Seelman et al. 2017). While the culture around the LGBTQIA+ community has demonstrated a broadening acceptance, those living in rural areas may continue to feel isolated and face more challenges. This can lead to a larger impact on their mental health due to limited resources for support or professional care. In the transgender population, lifetime prevalence of at least one suicide attempt is between 30% and 80% compared to 5% of the general population (Cogan et al. 2021). One study revealed that over a period of 1 year, 53% of transgender youth had symptoms of depression for at least 2 weeks compared to 30% of cisgender youth (Hall et al. 2019). Additional research has also shown that using one's chosen name can be associated with reductions in negative health outcomes in gender‐diverse youth (Pollitt et al. 2019). For Jamie, being continuously called by her given name by family, friends, and teachers served as a challenging reminder of the person she did not identify with, thereby increasing her distress and mental health symptoms.

Family acceptance or rejection during the adolescent period significantly impacts the mental health of adolescents. Jamie, who had not disclosed her identity to anyone, lacked family and social support, which led her to withdraw, negatively affecting her mental health both socially and academically. Adolescents from affectionate homes are more secure, trusting, and tolerant, and are less hostile (Aymerich et al. 2018). Family support fosters safe and trusting relationships and is imperative for adolescents who are questioning their gender identity. LGBTQIA+ youth with family acceptance experience reduced anxiety, depression, and suicidality as well as lower risk of sexual victimization and self‐harm behaviors (Call et al. 2021). Adversely, adolescents who report low family acceptance are 20 times more likely to report depression and 56.8% more likely to have a suicide attempt in their lifetime (Ryan et al. 2010). Families rely on one another for social and emotional needs while health care providers play an important role in helping mitigate relationships and guide families to needed resources (Bhattacharya et al. 2021). Assessing gender identity and gaining insight into family dynamics enables providers to foster a stronger support system for both the adolescent and the family system as a whole.

Currently, there is no national criterion for providers or clinicians to guide the collection of gender identity data in the health care environment for adolescents (Progovac et al. 2018). However, advocacy groups, such as the National Academy of Medicine and The Joint Commission on Healthcare, all offer guidelines and recommendations specific to routine documentation of gender identity to facilitate efforts in the reduction of health disparities within the transgender and gender diverse community (Maragh‐Bass et al. 2017). In addition, AAP (American Academy of Pediatrics) released a policy statement in 2018 urging providers and caregivers to offer support and care for transgender and gender‐diverse children and adolescents (American Academy of Pediatrics 2018). Moreover, in 2019, the World Health Organization (WHO) removed transgender as a mental health diagnosis, recognizing the importance of affirming care (World Health Organization 2019).

As healthcare settings across the country move to align with the 2018 recommendations set forth by AAP, it is important to pause and evaluate the current state of gender inclusivity. Furthermore, strategies must be developed to more firmly embrace gender supportive healthcare within clinical settings. With that approach in mind and in keeping with best practice guidelines and recommendations, a program evaluation was performed to understand the degree to which a practice setting is aligned with the 2018 AAP recommendations. The AAP policy statement encourages the inclusion of gender identity in electronic health records, preferred name, and pronouns separately from their legal name and sex assigned at birth (American Academy of Pediatrics 2018). Given the lack of standardized protocols for gender identity documentation, despite recommendations from organizations such as the AAP and Joint Commission, and the limited literature assessing compliance in clinical settings, this program evaluation offers an important contribution. It provides new insights to guide discussions, highlights gaps for further exploration, and identifies actionable responses needed to improve outcomes and care for adolescents in mental health settings as they relate to gender identity documentation.

## 3 Methods

The program evaluation was conducted in an outpatient mental health clinic in a rural area. The clinic provides comprehensive mental health services to both adults and pediatric patients. The multidisciplinary team consists of two full‐time Advanced Practice Providers (APPs), one part‐time telemedicine Medical Doctor, two registered nurses, in addition to case managers, therapists, and multiple support staff to include peer workers. All adolescents have access to a designated Human Rights Advocate and mental health treatment services regardless of age, assigned sex at birth, gender, ethnicity, and race. Financial means are neither a consideration nor a barrier to treatment services. All health care services provided are voluntary, and client autonomy is respected and with an emphasis on beneficence by promoting well‐being to improve mental health. As part of the evaluation process, stakeholders, including clinical staff, case managers, and administrative leaders, were engaged. In addition, a regional assessment was conducted to capture the local needs, resources, and barriers unique to the rural setting.

The program evaluation design focused specifically on the documentation of gender identity by assessing the consistency of gender documentation on the face sheet form included with the admission intake paperwork within the electronic health record (EHR) of adolescent charts.

A retrospective chart review of quantitative data was conducted; data retrieval and collection included the EHRs of children and adolescents aged 4–18 years old. From 2020 to 2023, a total of 151 children and adolescents, aged 4–18 years old, were treated at the clinic. The gender distribution was as follows: 39% female (*n* = 59), 51.7% male (*n* = 78), 1.3% non‐conforming (n = 2), and 7.9% (*n* = 12) undocumented gender.

## 4 Results

In 2020, clinic census totaled 3 patients, with no gender identity having been documented. In 2021, clinic census totaled 3 patients, with gender identity documented for 1 patient (33.3%). In 2022, upon resuming full operations after following COVID‐19‐associated closure, the clinic admitted and treated 44 patients, with 95.5% (*n* = 42) having documented gender identities. In 2023, a total of 101 patients were treated, with 95% (*n* = 96) having documented gender identities. Significant improvement in gender identity documentation was noted from 0% to 95%, over the 4‐year time period from 2020 to 2023, which could be suggestive of a shift in culture as well as societal awareness and acceptance of diverse gender identities.

Currently, gender identity is assessed only during the initial intake at the time of admission. However, this approach does not account for potential changes or developments in an adolescent's gender identity over time. For instance, while Jamie knew she was transgender, she did not feel comfortable disclosing this to the nurse she had never met, especially with her mother present during the intake and not knowing the nurse. Without periodic reassessments, Jamie—and others in similar situations—may never feel safe or comfortable enough to share this important information, leading to ongoing inadequate support and care services and recognition of her gender identity.

Additionally, one's gender identity may not always align with pronouns. Therefore, pronouns should be assessed and documented separately, as assuming pronouns correlate with gender identity can lead to inaccuracies (Olson et al. 2022). Establishing uniformity in admission assessment processes, inclusive of gender identity, can have a significant impact on improving care. Incorporating formal policy into the annual review training will help ensure staff stay up to date. Research suggests that limited exposure to gender non‐conforming clients can affect how providers assess gender identity (Sedlak and Boyd 2016). Hence, the formation of a diversity committee could facilitate and help maintain best practice standards and ensure continuous improvement for both clinical providers and support staff.

## 5 Conclusion

Supporting gender‐diverse adolescents in building trust within clinics and communities improves client outcome (Hope et al. 2022). Jamie's experience reflects what many gender‐diverse adolescents face daily, feeling unseen and unrecognized. Mental health settings can address this by prioritizing gender identity documentation. Adherence to AAP guidelines help create safe, affirming environments where vulnerable population can receive care and support, they need. Leaders who model inclusivity set the tone for respectful, transparent care, beginning with consistent gender identity documentation.

This program evaluation demonstrates that systemic gender identity documentation is feasible in rural outpatient clinics. While generalizability is limited to a single site, the findings are relevant to other settings where resources are constrained but inclusive care is essential. The results provide a low‐cost model for clinics that can provide a broader initiative to advance equity for gender‐diverse youth. Collaborations across outpatient settings can refine best practices, expand outcomes research, and guide policies that promote inclusive care across rural and urban systems. Findings of this program evaluation can help provide guidance and insights. Evaluating clinical settings and establishing formal protocols for gender identity documentation can help ensure consistent compliance and affirming care. These findings can also guide public health nurses in reducing disparities, influencing policy, and supporting multi‐step efforts to improve care for gender‐diverse youth.

## Funding

The authors have nothing to report.

## Conflicts of Interest

The authors declare no conflicts of interest.

## Data Availability

The data that support the finding of this program evaluation are not available due to privacy and institutional restrictions.
